# Supplementary material for: Inequities in access to primary care among opioid recipients in Ontario, Canada: A population-based cohort study
Source: PLoS Med. 2021 Jun 1;18(6):e1003631. doi: 10.1371/journal.pmed.1003631 (PMC8168863; doi:10.1371/journal.pmed.1003631)
Supplement: S1 Fig — (DOCX) [file pmed.1003631.s002.docx]

**S1 Fig. Tests of Proportional Hazards Assumptions**

1. Primary Analysis:


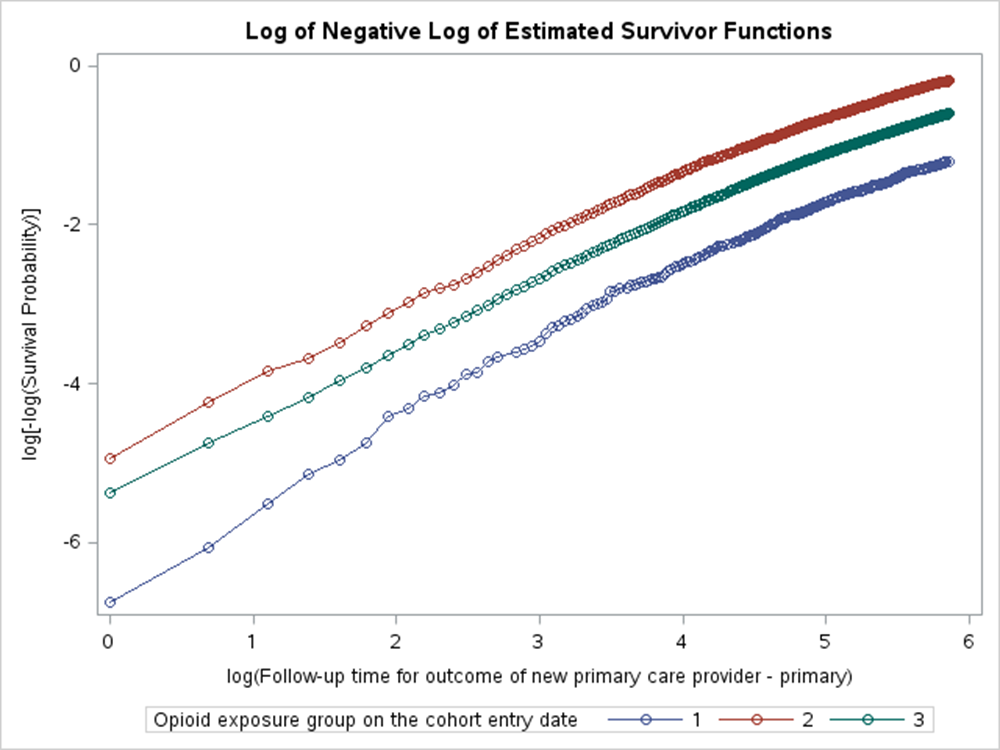


1. Sensitivity Analysis 1: First instance of primary care attachment:


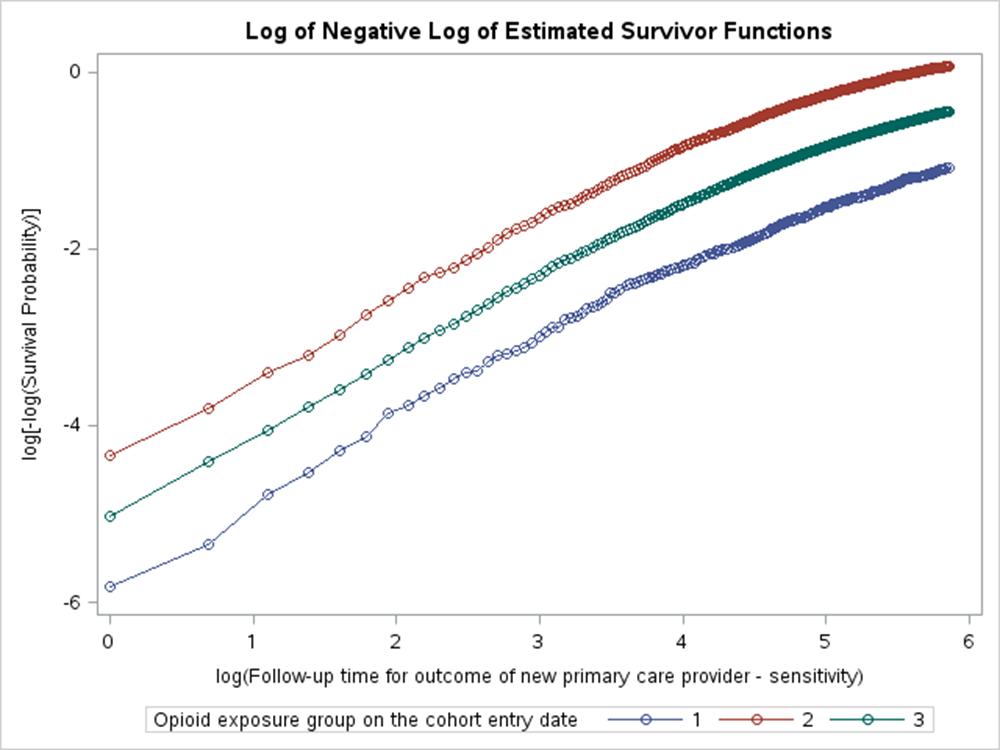


1. Sensitivity Analysis 2: Attachment to PEM

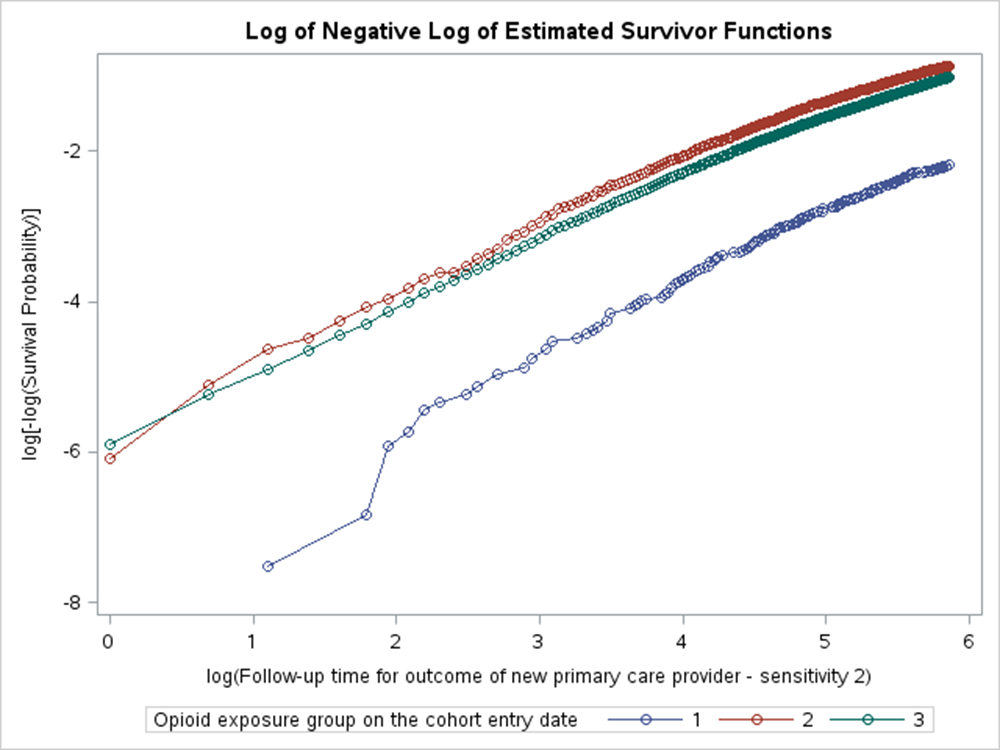

   Note: PH assumption confirmed by checking time-varying exposure: p-value =0.10
2. Sensitivity Analysis 3: Attachment to PEM or CHC

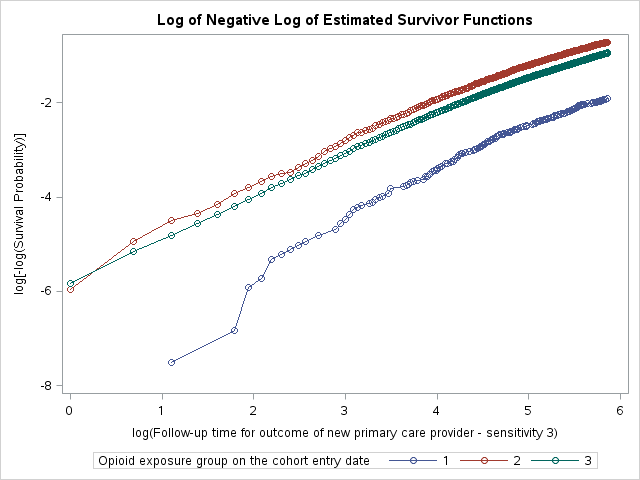

   Note: PH assumption confirmed by checking time-varying exposure: p-value =0.30

**Figure S2. Rates of emergency department visits during the one year prior to loss of primary care attachment and during the period without primary care attachment, by opioid exposure group**

**Figure S3. Rates of health services use for opioid toxicity during the one year prior to loss of primary care attachment and during the period without primary care attachment, by opioid exposure group**
