# Supplementary material for: Inequities in access to primary care among opioid recipients in Ontario, Canada: A population-based cohort study
Source: PLoS Med. 2021 Jun 1;18(6):e1003631. doi: 10.1371/journal.pmed.1003631 (PMC8168863; doi:10.1371/journal.pmed.1003631)
Supplement: S2 Fig — (DOCX) [file pmed.1003631.s003.docx]

**S2 Fig. Rates of emergency department visits during the one year prior to loss of primary care attachment and during the period without primary care attachment, by opioid exposure group**
