# Supplementary material for: Inequities in access to primary care among opioid recipients in Ontario, Canada: A population-based cohort study
Source: PLoS Med. 2021 Jun 1;18(6):e1003631. doi: 10.1371/journal.pmed.1003631 (PMC8168863; doi:10.1371/journal.pmed.1003631)
Supplement: S3 Fig — (DOCX) [file pmed.1003631.s004.docx]

**S3 Fig. Rates of health services use for opioid toxicity during the one year prior to loss of primary care attachment and during the period without primary care attachment, by opioid exposure group**
