# Supplementary material for: Inequities in access to primary care among opioid recipients in Ontario, Canada: A population-based cohort study
Source: PLoS Med. 2021 Jun 1;18(6):e1003631. doi: 10.1371/journal.pmed.1003631 (PMC8168863; doi:10.1371/journal.pmed.1003631)
Supplement: S1 Text — (DOCX) [file pmed.1003631.s005.docx]

**Table A. Diagnostic and billing codes and data sources used to define clinical covariates**

| **Covariate** | **Data Source** | **Codes** |
| --- | --- | --- |
| **Alcohol-use disorder** | Emergency department visit (all diagnosis types), inpatient  hospitalization (all diagnosis  types), or physician claim in the 3 years prior to opioid initiation | **ICD-10:** F10, G31.2, G62.1, G72.1, I42.6, K29.2, K70, K86.0, Z50.2, Z71.4, Z86.40 |
|  |  | **OHIP DX:** 291, 303 |
| **Asthma** | Asthma ICES-derived cohort | **ICD-10:** J45, J46 |
|  |  | **OHIP DX:** 493 |
| **Chronic kidney disease** | Emergency department visit (all diagnosis types), inpatient  hospitalization (all diagnosis  types), or physician claim in the 3 years prior to opioid initiation | **ICD-10:** E10.2, E11.2, E13.2, E14.2, I12, I13, N08, N18, N19 |
|  |  | **OHIP DX:** 403, 585 |
| **COPD** | COPD ICES-derived cohort | **ICD-10:** J41, J42, J43, J44 |
|  |  | **OHIP DX:** 491, 492, 496 |
| **Diabetes** | Diabetes ICES-derived cohort | **ICD-10:** E10, E11, E13, E14 |
|  |  | **OHIP DX:** 250 |
|  |  | **ODB:** Claim for insulin or oral anti-glycemics |
| **Liver disease** | Emergency department visit (all diagnosis types), inpatient  hospitalization (all diagnosis  types), or physician claim in the 3 years prior to opioid initiation | **ICD-10:** K70, K71, K72, K73, K74, K75, K76, K77 |
|  |  | **OHIP DX:** 571 |
| **Opioid-related emergency department visits and hospitalizations** | Emergency department visit (all diagnosis types), or inpatient  hospitalization (admission diagnosis) | **ICD-10:** T40.0, T40.1, T40.2, T40.3, T40.4, T40.6 |
| **Primary care core services** | Physician claims in OHIP | **OHIP billing codes:** A001, A002, A003, A007, A903, E075, G212, G271, G372, G373, G365, G538, G539, G590, G591, K005, K013, K017, P004, K130, K131, K132, K030 |
| **Mental health and substance use disorder diagnoses (definitions leveraged from the Ontario Mental Health and Addictions Scorecard and Evaluation Framework)** | | |
| **Anxiety disorders** | Emergency department visit (main problem), inpatient  hospitalization (most responsible diagnosis), or mental health hospitalization (primary discharge diagnosis, or provisional diagnosis if primary discharge diagnosis was missing) in the 3 years prior to opioid initiation | **ICD-10:** F40, F41, F42, F43, F48.8, F48.9, F93.1, F93.2 |
|  |  | **DSM-IV:** 300.0, 300.2, 300.3, 308.3, 309.0, 309.24, 309.28, 309.3, 309.4, 309.8, 309.9. Provisional: 7, 15 |
| **Deliberate self-harm** | Emergency department visit (secondary problem field) or inpatient  hospitalization (secondary diagnosis) in the 3 years prior to opioid initiation | **ICD-10:** X60-X84, Y10-Y19, Y28 AND main problem or most responsible diagnosis NOT F06-F99 |
| **Mood disorders** | Emergency department visit (main problem), inpatient  hospitalization (most responsible diagnosis), or mental health hospitalization (primary discharge diagnosis, or provisional diagnosis if primary discharge diagnosis was missing) in the 3 years prior to opioid initiation | **ICD-10:** F30, F31, F32, F33, F34, F38, F39, F53.0 |
|  |  | **DSM-IV:** 296, 300.4, 301.13, 311. Provisional: 6 |
| **Schizophrenia** | Emergency department visit (main problem), inpatient  hospitalization (most responsible diagnosis), or mental health hospitalization (primary discharge diagnosis, or provisional diagnosis if primary discharge diagnosis was missing) in the 3 years prior to opioid initiation | **ICD-10:** F20 (excluding F20.4), F22, F23, F24, F25, F28, F29, F53.1 |
|  |  | **DSM-IV:** 295, 297, 298. Provisional: 5 |
| **Substance-related disorders** | Emergency department visit (main problem), inpatient  hospitalization (most responsible diagnosis), or mental health hospitalization (primary discharge diagnosis, or provisional diagnosis if primary discharge diagnosis was missing) in the 3 years prior to opioid initiation | **ICD-10:** F10, F11, F12, F13, F14, F15, F16, F17, F18, F19, F55 |
|  |  | **DSM-IV**: 291 (excluding 291.82), 292 (excluding 292.85), 303, 304, 305. Provisional: 4 |
| **Other mental health disorders** | Emergency department visit (main problem), inpatient  hospitalization (most responsible diagnosis), or mental health hospitalization (primary discharge diagnosis, or provisional diagnosis if primary discharge diagnosis was missing) in the 3 years prior to opioid initiation | **ICD-10:** Any mental health disorder was defined as a main problem, most responsible diagnosis, or primary discharge diagnosis of F06-F99, OR secondary problem or diagnosis of X60-X84, Y10-Y19, Y28 when the main problem, most responsible diagnosis, or primary discharge diagnosis was NOT F06-F99.  Other mental health disorders were defined as anyone flagged with a mental health disorder as defined above, but not an indication of an anxiety disorder, deliberate self-harm, mood disorder, schizophrenia, or substance-related disorder. |
|  |  | **DSM-IV**: Any mental health disorder was defined as any DSM-IV code (excluding 290, 294, and a provisional diagnosis of 2 when the primary discharge diagnosis was missing).  Other mental health disorders were defined as anyone flagged with a mental health disorder as defined above, but not an indication of an anxiety disorder, deliberate self-harm, mood disorder, schizophrenia, or substance-related disorder. |

**Abbreviations:** COPD – chronic obstructive pulmonary disease, DSM-IV – Diagnostic and Statistical Manual of Mental Disorders, 4th Edition, Text Revision, HIV – human immunodeficiency virus, ICD-10 – International Classification of Diseases, 10th Revision, ODB – Ontario Drug Benefit Claims Database, OHIP DX – Ontario Health Insurance Plan diagnostic codes

**Table B.** **Opioid-related characteristics among individuals in the study cohort receiving opioid agonist therapy or chronic opioid therapy**

| **Receiving opioid agonist therapy (N=1,727)** | **Receiving methadone** | 1,323 (76.6%) |
| --- | --- | --- |
|  | **Receiving buprenorphine/naloxone** | 408 (23.6%) |
|  | **Most recent opioid agonist therapy prescription prescribed by rostering physician** | <5 |
|  | **Number of days of methadone therapy in prior 14 days (Median, IQR)** | 14 (13-15) |
|  | **Number of days of buprenorphine/naloxone therapy in prior 14 days (Median, IQR)** | 14 (12-22) |
| **Receiving long-term opioid pain therapy (N=3,644)** | **Opioid formulation for dispensed prescription(s) overlapping enrolment end date** |  |
|  | Long-acting | 787 (21.6%) |
|  | Immediate release | 1,830 (50.2%) |
|  | Both long-acting and immediate release | 891 (24.5%) |
|  | No opioid overlapping enrolment end | 136 (3.7%) |
|  | **Average daily dose for dispensed prescription(s) overlapping the enrolment end date – Median (IQR)** | 45 (23-120) |
|  | **More than 1 prescriber for opioid prescriptions dispensed in the 100 days prior to/including enrolment end date – N (%)** | 854 (23.4%) |

Values are N (%) unless otherwise specified

Abbreviations: IQR – interquartile range

**Table C.** **Source of primary care among individuals in the study cohort who became attached within one year of primary care provider loss**

| **Opioid exposure** | **Primary care attachment** | **Primary care enrolment model attachment** | **Community health centre**  **attachment** | **Fee-for-service attachment** |
| --- | --- | --- | --- | --- |
| **Overall** | 65,691 | 48,605 (74.0%) | 2,858 (4.4%) | 14,228 (21.7%) |
| **Opioid unexposed individuals** | 63,232 | 47,086 (74.5%) | 2,656 (4.2%) | 13,490 (21.3%) |
| **Receiving opioid agonist therapy** | 450 | 197 (43.8%) | 54 (12.0%) | 199 (44.2%) |
| **Receiving long-term opioid pain therapy** | 2,009 | 1,322 (65.8%) | 148 (7.4%) | 539 (26.8%) |

Values are N (%) unless otherwise specified

**Table D. Type of primary care enrolment model among individuals in the study cohort who became attached to an enrolment model within one year of primary care provider loss**

| **Opioid exposure** | **Primary care enrolment model attachment** | **Capitation model** | **Enhanced fee-for-service model** | **Other model** |
| --- | --- | --- | --- | --- |
| **Overall** | 48,605 | 32,539 (67.0) | 15,345 (31.6) | 721 (1.5) |
| **Opioid unexposed individuals** | 47,086 | 31,519 (66.9) | 14,880 (31.6) | 687 (1.5) |
| **Receiving opioid agonist therapy** | 197 | 113-117** | 79 (40.1) | <5 |
| **Receiving long-term opioid pain therapy** | 1,322 | 903-907** | 386 (29.2) | 29-33** |

Values are N (%) unless otherwise specified

**Values censored (ranges provided) to avoid residual disclosure of cell counts ≤5
