# Supplementary material for: Inequities in access to primary care among opioid recipients in Ontario, Canada: A population-based cohort study
Source: PLoS Med. 2021 Jun 1;18(6):e1003631. doi: 10.1371/journal.pmed.1003631 (PMC8168863; doi:10.1371/journal.pmed.1003631)
Supplement: S1 Protocol — (DOCX) [file pmed.1003631.s006.docx]

| Note: Any changes to the original protocol are underlined and prefaced using the heading “Post-hoc after reviewer comments:” | | |
| --- | --- | --- |
| Project Cohort | | |
| **Study Design** | Cohort study | |
| **Cohort** | - Identify Ontario residents alive and eligible for the Ontario Health Insurance Plan (OHIP) on December 31, 2015   - Death date in Registered Persons Database missing or after December 31, 2015   - Birth date in Registered Persons Database prior to or on December 31, 2015   - Ontario resident in Registered Persons Database on December 31, 2015   - Eligible for OHIP on December 31, 2015 - Further identify people meeting the above criteria who were also rostered to a primary care provider on December 31, 2015, and whose relationship with their provider ended between January 1, 2016 and December 31, 2017   - Identify individuals in the Client Agency Program Enrolment (CAPE) database (which contains people enrolled in a primary care enrolment model and dates of enrolment) who were rostered to a primary care provider on December 31, 2015   - Among those rostered to a primary care provider on December 31, 2015, identify individuals who had an ‘ENDCAPE’ date between January 1, 2016 and December 31, 2017, indicating that their enrolment with the physician they were rostered to on December 31, 2015 ended between January 1, 2016 and December 31, 2017. Classify these individuals according to their opioid exposure (defined below) on the date of primary care provider loss | |
| **Study Period** | - **Reference date to identify rostered individuals**: December 31, 2015 - **Accrual of individuals who lose their primary care provider**: January 1, 2016 – December 31, 2017 - **Period during which to exclude anyone who died or had an outcome**: Within the 14 days following date of primary care provider loss - **Cohort entry date**: 14 days following date of primary care provider loss - **Follow-up from cohort entry until:**   - Outcome (defined below)   - Death   - 351 days following the cohort entry date   - End of study period (December 31, 2018) | |
| **Exclusions (in order)** | *Step* | Description |
|  | 1 | Age <18 or >105 on date of primary care provider loss |
|  | 2 | Date of last health care contact (DOLC) falls prior to 8 years before the date of primary care provider loss |
|  | 3 | Died prior to or on the primary care provider loss date |
|  | 4 | Resided out of province on the primary care provider loss date |
|  | 5 | Not eligible for OHIP on the primary care provider loss date |
|  | 6 | Terminated for reasons other than physician ended patient enrolment, physician ended enrolment because patient moved out of physician’s catchment area, physician ended enrolment per patient request, or enrolment terminated by patient (i.e., CAPE ‘TERMINATION’ not in (‘44’, ‘53’, ‘56’, ‘57’)) |
|  | 7 | Less than 1 year of CAPE enrolment prior to primary care provider loss |
|  | 8 | Re-enrolled in CAPE with physicians from the same group as the previous rostering physician in any period of time after primary care provider loss |
|  | 9 | Had an OHIP claim with the previous rostering physician in any period of time after primary care provider loss |
|  | 10 | Died within the 14 days following primary care provider loss |
|  | 11 | Experienced primary care attachment within the 14 days following primary care provider loss |
|  | 12 | In exposure group 1 (defined below) and also receiving chronic opioid therapy |
|  | 13 | In exposure group 2 (defined below) with an opioid agonist therapy (OAT) prescription dispensed in the year prior to or including primary care provider loss |
|  | 14 | Not defined in exposure group 1 or exposure group 2 (defined below), but has a history of opioid dispensing or health services use for opioid toxicity in the 3 years prior to or including primary care provider loss |
|  | 15 | Received palliative care services in the 1 year prior to/including date of provider loss:   - Any CIHI-DAD hospitalization with patient service (PATSERV) = 58. **OR** - Any OHIP billing with one of the following billing codes: OHIP FEECODE A945, B998, C945, C882, C982, K023, W872, W882, W972 or W982. **OR** - Any ALR record (use Visit_Date variable) with one of the following:   - Visit_Program_Code=PA (ALR Clinic Visit dataset)   - Intent_of_systemic_treatment=P (ALR Systemic dataset)   - Intent_of_radiation=P (ALR Radiation dataset) |

| Variable Definitions | | |
| --- | --- | --- |
| **Main Exposure** | **Among the cohort, assign individuals to one of three groups based on their opioid exposure on the date of primary care provider loss:**   1. People recently dispensed a prescription for opioid agonist therapy (OAT; methadone or buprenorphine/naloxone)    - To identify prescriptions for OAT, use the ODPRN NMS master druglist to identify drug identification numbers (DINs) where NMS_GROUP=‘OPIOID’ and OMT=‘Y’. Use dclass=‘METHADONE_OMT’ for methadone, and dclass=‘BUPRENORPHINE’ for the combination product buprenorphine/naloxone    - Use NMS to capture any methadone claim dispensed in the 14 days prior to or including the date of primary care provider loss    - Use NMS to extract buprenorphine/naloxone claims dispensed in the 30 days prior to or including the date of primary care provider loss that also have a days’ supply overlapping the 14 days prior to or including the date of primary care provider loss      1. We checked the days’ supply of buprenorphine/naloxone prescriptions dispensed in the 90 days prior to or including the date of primary care provider loss and found that 99.27% had a days’ supply of 14 or less, and 99.93% had a days’ supply of 30 or less. 30 days was therefore chosen as the lookback period since almost all dispensed buprenorphine/naloxone prescriptions have a days’ supply within this period    - Exclude individuals meeting the above criteria that are also captured in group 2 below (exclusion 12)    - Individuals meeting these criteria will be defined as exposure group 1 – OAT recipients 2. People receiving long-term therapy with opioids for pain    - To identify prescription opioids for pain, use the ODPRN NMS master druglist to identify DINs where NMS_GROUP=‘OPIOID’, OMT=‘N’, dclass^=(‘EXCL’, ‘COUGH’), and dclass_broad^=(‘RUNS’, ‘MAID’).    - Use NMS to capture prescription opioid claims dispensed in the 190 days prior to or including the date of primary care provider loss (so that there is a 100 day period to review use of prescription opioids, and 90 days prior to that to assess prior prescriptions dispensed that overlap the 100 day review period)    - People receiving long-term therapy with opioids for pain will be defined as individuals who had 90 unique days covered with prescription opioid use in the 100 days prior to or including the date of provider loss, determined based on days’ supply on claims dispensed in the 190 day period    - Exclude individuals with an OAT prescription dispensed in the year prior to or including the date of provider loss (exclusion 13)    - Individuals who meet these criteria will be defined as exposure group 2 – long-term opioid pain therapy recipients 3. People without a history of opioid dispensing or health services use for opioid toxicity in the 3 years prior to or including the date of provider loss    - To identify claims for prescription opioids for any indication, use the ODPRN NMS master druglist to identify DINs where NMS_GROUP=‘OPIOID’    - Use NMS to capture prescription opioid claims dispensed to individuals in the study cohort in the 3 years prior to or including the date of primary care provider loss    - Use DAD and NACRS to identify emergency department (ED) visits and hospitalizations for opioid toxicity among individuals in the 3 years prior to or including the date of primary care provider loss (See [Appendix A](#_Appendix_A:_Codes) for diagnosis codes)    - Individuals **without** a claim for a prescription opioid **AND** **without** an ED visit or hospitalization for opioid toxicity in the 3 years prior to or including primary care provider loss will be defined as exposure group 3 |  |
| **Primary Outcome Definition** | The primary outcome is securing a new primary care provider, and is a hierarchical, composite outcome consisting of:   - 1) First instance of re-enrolment with a primary care provider in CAPE, 2) first indication of 3 or more visits with a primary care provider (physician or nurse practitioner) through a community health centre (CHC), or 3) first indication of 3 or more visits with a comprehensive primary care physician in OHIP, within 351 days following the cohort entry date (i.e., within 1 year of primary care provider loss date)   - To identify individuals re-enrolled in CAPE, link the cohort to the CAPE database to identify records for the same individuals where the ‘STRTCAPE’ date is greater than the cohort entry date     - There may be multiple records for a given individual, so select the record with the earliest ‘STRTCAPE’ date following the cohort entry date   - To identify visits with a primary care provider outside of CAPE:     - **CHC:**       - Link individuals in the study cohort to the CHC patients dataset. Identify CHC encounters for these individuals over 1.5 years after cohort entry, and then link these encounters to the CHC providers dataset. Restrict to encounters with physicians or nurse practitioners. Identify individuals who had at least three visits to a CHC with a physician or nurse practitioner during the follow-up period. Among these people, select the record with the earliest encounter date following the cohort entry date.     - **OHIP**:       - Extract core primary care billing claims for individuals in the study cohort over 1.5 years after cohort entry (see core primary care billing codes in [Appendix A](#_Appendix_A:_Codes)).       - Link the physicians on the OHIP billing claims to ICES Physician Database (IPDB) to determine practice type (‘PRACTYPE’ variable). Identify individuals who had at least three core primary care OHIP claims billed by a physician with a practice type of comprehensive primary care. Among these people, select the record with the earliest encounter date following the cohort entry date. - Using the definitions above, determine whether individuals experienced primary care attachment in CAPE, and identify the date they were enrolled. Among individuals who were not enrolled in CAPE, consider attachment in CHCs, and identify the date of the first encounter. Among individuals who were not enrolled in CAPE or attached to a CHC, consider attachment through OHIP claims and identify the date of the first claim. - Censor primary care attachment on death, 351 days following the cohort entry date, or the end of study period (December 31, 2018) |  |
| **Secondary Outcome Definition** | - Number and rate of emergency department visits during the period without a primary care provider   - Use NACRS to identify visits that occurred following the cohort entry date but prior to the end follow-up date for the primary outcome. Count the number of visits per person   - Denominator will be follow-up time for the primary outcome |  |
| **Tertiary Outcome Definition** | - Number and rate of emergency department visits and hospitalizations for opioid toxicity during the period without a primary care provider   - See [Appendix A](#_Appendix_A:_Codes) for diagnosis codes   - Identify incidents that occurred following the cohort entry date but prior to the end follow-up date for the primary outcome. Count the number of incidents per person   - Denominator will be follow-up time for the primary outcome |  |
| **Baseline Characteristics** | **Report the number of people in the cohort, and the following baseline characteristics, by the 3 exposure groups:**   - Age – RPDB age variable (reference to the date of primary care provider loss)   - Median (IQR)   - Age group (n, %)     - 18-24     - 25-34     - 35-44     - 45-64     - 65+ - Sex (n, %) – RPDB sex variable - Urban/rural location of residence (n, %) – RPDB rural variable (reference to the date of primary care provider loss; include as categorical variable with missing as a separate category) - Residence in northern Ontario (n, %) – RPDB LHIN variable (reference to the date of primary care provider loss)   - Northern Ontario defined as individuals residing in LHINs 13 or 14 - Income quintile (n, %) – RPDB incquint variable (reference to the date of primary care provider loss; include as categorical variable with missing as a separate category) - Ontario Marginalization Index quintiles (n, %)   - Dependency – Q1-Q5 and missing   - Material Deprivation – Q1-Q5 and missing   - Ethnic Concentration – Q1-Q5 and missing   - Residential Instability – Q1-Q5 and missing - Diabetes diagnosis prior (excluding index) to date of primary care provider loss (use the ICES-derived cohort: Ontario Diabetes Dataset) (n, %) - COPD diagnosis prior (excluding index) to date of primary care provider loss (use the ICES-derived cohort: Chronic Obstructive Pulmonary Disease) (n, %) - Asthma diagnosis prior (excluding index) to date of primary care provider loss (use the ICES-derived cohort: Ontario Asthma dataset) (n, %) - Health service utilization for liver disease in the 3 years prior (excluding index) to date of primary care provider loss (n, %)   - See [Appendix A](#_Appendix_A:_Codes) for diagnosis codes - Health service utilization for chronic kidney disease in the 3 years prior (excluding index) to date of primary care provider loss (n, %)   - See [Appendix A](#_Appendix_A:_Codes) for diagnosis codes - Health service utilization for alcohol use disorder in the 3 years prior (excluding index) to date of primary care provider loss (n, %)   - See [Appendix A](#_Appendix_A:_Codes) for diagnosis codes - ED or hospitalization visit for opioid toxicity in the 3 years prior (excluding index) to date of primary care provider loss (n, %)   - See [Appendix A](#_Appendix_A:_Codes) for diagnosis codes - Any ED visit or hospitalization for mental health and addictions diagnoses in the 3 years prior (excluding index) to date of primary care provider loss   - NACRS     - Using the NACRS_ED_EPI dataset (which links ED records into episodes), identify mental health-related emergency department visits that occurred in the 3 years prior to the date of primary care provider loss using the ‘REGDATE’ variable. Restrict to visits where the ‘MHASEF_OLD’ variable ne ‘’, and use this variable to determine mental health and addictions diagnosis categories   - DAD and OMHRS     - Using the DADOMHRS_EPI_MHA dataset (which contains episodes of care comprised of BOTH acute inpatient hospitalizations and psychiatric admissions), identify mental health-related hospitalizations that occurred in the 3 years prior to date of primary care provider loss using the ‘EPI_DDATE’ variable (exclude those without a discharge date). Restrict to visits where the ‘MHASEF_OLD’ variable ne ‘’, and use this variable to determine mental health and addictions diagnosis categories - Enrolment program type (CAPE ‘PROGTYPE’ variable)   - Collapse into Capitation (FHO/FHN), Enhanced Fee-for-Service (FHG/CCM), or Other (all other) - End reason code for patient enrolment (CAPE ‘TERMINATION’ variable) - Health system utilization in the 1 year prior (excluding index) to date of primary care provider loss   - Number of physician visits **for any reason** (Mean + SD)     - Extract visits from OHIP, restrict to visits with a location of Office, Home, LTC, Phone, or Undefined. Count one claim per person per physician per day   - Number of physician visits **for any reason** with CAPE physician (Mean + SD)     - Extract visits from OHIP, restrict to visits with a location of Office, Home, LTC, Phone, or Undefined and those with the physician that they were previously rostered to. Count one claim per person per day   - Number of ED visits (Mean + SD)     - Extract visits from NACRS, and count the number of visits per person   - Number of inpatient hospitalizations (Mean + SD)     - Extract visits from DAD     - Count the number of unique hospital episodes per person (epi variable in DAD) - John’s Hopkins Aggregated Diagnosis Groups (ADGs) (see [Appendix B](#_Appendix_B:_John’s))   - Pull OHIP data in the 2 years prior (excluding index) to the date of primary care provider loss. Exclude X-ray claims   - Pull DAD/SDS data in the 2 years prior (excluding index) to the date of primary care provider loss   - Run ACG macro. Obtain the ADGs from the macro output. Sum the number of ADGs per person and categorize as:     - Non-users/no or only unclassified diagnoses     - 1-2     - 3-4     - 5-6     - 7+ - Prescriptions dispensed in the 1 year prior (excluding index) to date of primary care provider loss. Use the ODPRN NMS master druglist to identify the following types of prescriptions, and extract claims from NMS:   - Stimulants (n, %)     - NMS_group = “STIM”   - Benzodiazepines (n, %)     - NMS_group = “BZD”   - Synthetic THC products     - NMS_group= “THC” - For individuals in exposure group 1 only:   - Report the N (%) dispensed methadone vs. buprenorphine/naloxone in the lookback period prior to or on the date of primary care provider loss   - Report the N (%) for whom the most recent OAT prescription was prescribed by the rostering physician   - **Post-hoc after reviewer comments:** Report the days’ supply dispensed in the 14 days prior to or including the date of primary care provider loss, for people dispensed methadone vs. buprenorphine/naloxone - For individuals in exposure group 2 only:   - Report the median (IQR) opioid dose on the date of provider loss     - Identify all dispensed prescriptions for opioids for pain overlapping with the date of provider loss, and calculate the average daily dose in mg morphine equivalent on that date   - Report the type of opioid formulation on the date of provider loss (N, %)     - Identify all dispensed prescriptions for opioids for pain overlapping with the date of provider loss. Among these prescriptions, identify the opioid formulation: long-acting, immediate release agent, or both, or no opioid overlapping enrolment loss date. Use the LA variable from the ODPRN NMS master druglist to form the first three categories   - Report the median (IQR) number of prescribers for opioid prescriptions dispensed in last 100 days     - Use NMS to capture prescription opioid claims dispensed in the 100 days prior to or including the date of provider loss, and count the number of unique prescribers. Report Median (IQR)     - **Post-hoc after reviewer comments:** Change this to report the N (%) of people with more than 1 prescriber identified on opioid prescriptions dispensed in the 100 days prior to or including the provider loss date |  |

| Analysis Plan |
| --- |
| **Statistical Analyses** |
| **Outcome 1: First instance of re-enrolment with a primary care provider in CAPE, or indication of attachment with a non-CAPE primary care provider in CHC or OHIP**   - - Generate Kaplan-Meier survival curves for the outcome     - Report results of the log-rank test to compare the survival curves     - Report median (IQR) time to rostering with a new primary care provider in all groups, and the overall rates (per 1,000 person days) of rostering with a new primary care provider   - Run a Cox Proportional Hazards model (group 3 is the referent)     - Adjust for covariates that are unbalanced between the groups. For the Ontario Marginalization Index covariates, consider all 4 marginalization indices in the analysis.     - Report the HR and 95% CI. Also report the source of primary care provider, and for those enrolled in CAPE, the type of enrolment model, overall and by group   **Outcome 2: Number of emergency department visits during the period without a primary care provider**   - - Calculate and report the number of ED visits, person-days of follow-up, and rate of visits (per 1,000 person-days) in each exposure group during the period without a primary care provider.   - Calculate and report the number of ED visits, person-days of follow-up, and rate of visits (per 1,000 person-days) in each exposure group in the 1 year prior to provider loss (i.e., use the “number of ED visits” variable in the baseline characteristics section)   - Run a Poisson GEE model (using IKN as the cluster, since there will be two measurements for each person [i.e., number of ED visits in the 1 year prior to provider loss, and number of ED visits during the period without a provider]), stratified by opioid exposure group, to compare the rate of ED visits between the periods     - Adjust for covariates that are unbalanced. Consider all 4 marginalization indices in the analysis     - Report rate ratios and 95% CI   **Outcome 3: Number and rate of emergency department visits and hospitalizations for opioid toxicity during the period without a primary care provider**   - - Calculate and report the number of events, person-years of follow-up, and rate of events (per 1,000 person-days) in each exposure group during the period without a primary care provider.   - Calculate and report the number of events, person-years of follow-up, and rate of events (per 1,000 person-days) in each exposure group in the 1 year prior to provider loss   - Run a Poisson GEE model (using IKN as the cluster, since there will be two measurements for each person) stratified by opioid exposure group for groups 1 & 2, to compare the rate of opioid toxicity across the periods     - Adjust for covariates that are unbalanced. Consider all 4 marginalization indices in the analysis     - Report rate ratios and 95% CI     - **The fully adjusted model would not converge – decision was made to adjust for a limited number of covariates:** **age, sex, northern or southern region of residence, health services use for alcohol use disorder, emergency department visit or hospitalization for mental health diagnoses, number of outpatient visits to previous rostering physician, and number of hospitalizations in the year prior to provider loss, as determined via email exchange on Feb. 19^th^ 2020** |
| **Sensitivity Analyses:**   1. Repeat the analysis for outcome #1 by modifying the outcome to select the first date someone secured a primary care provider from any source, instead of using the hierarchical approach. Re-run the model with the same covariates. 2. Repeat the analysis for outcome #1 by modifying the outcome to consider primary care attachment as enrolment in CAPE only. Re-run the model with the same covariates. 3. Repeat the analysis for outcome #1 by modifying the outcome to consider primary care attachment as enrolment in CAPE or a CHC only. Re-run the model with the same covariates. 4. **Post-hoc after reviewer comments:** Repeat the analysis for outcome #1, with no modification of the outcome, but restrict to those whose enrolment termination reason was “Physician ended patient enrolment”. Re-run the model with the same covariates. 5. **Post-hoc after reviewer comments:** Repeat the analysis for outcome #1, by modifying the exposure variable to disaggregate the types of OAT into two categories: those who received buprenorphine/naloxone only, and those who received methadone only (exclude those who received both). Re-run the model with the same covariates. |
| **Tables/Figures** |
| **Table 1.** Table of baseline characteristics by exposure group with standardized differences |
| **Table 2.** Table of opioid-related baseline characteristics for those in the opioid agonist therapy and long-term opioid pain therapy groups |
| **Table 3.** Produce table with Kaplan-Meier survival statistics:   - Report median (IQR) time to rostering with a new primary care provider in all groups |
| **Figure 1.** Produce Kaplan-Meier survival curves for time to rostering with a new primary care provider |
| **Table 4.** Produce table with results of Cox proportional hazards model for rostering with a new primary care provider, and the overall and group-specific rates of rostering with a new primary care provider, for primary analysis and all sensitivity analyses for outcome #1 |
| **Table 5.** Produce table with the source of primary care provider for the primary hierarchical outcome, and for those enrolled in CAPE, the type of enrolment model, overall and by group |
| **Table 6.** Produce table with results of Poisson model for outcome #2 |
| **Table 7.** Produce table with results of Poisson model for outcome #3 |

# Appendix A: Codes for Covariates

| **Covariate** | **Data Source** | **Codes** |
| --- | --- | --- |
| Opioid toxicity | DAD, NACRS | **ICD-10-CA:**  T400-T404 or T406 |
| Alcohol Use Disorder | OHIP, DAD, NACRS | **OHIP Dxcode:**  291, 303  **ICD-10-CA:**  F10 K70 G312 G621 G721 I426 K292 K860 Z502 Z714 Z8640 |
| Chronic Kidney Disease | OHIP, DAD, NACRS | **OHIP Dxcode:**  403, 585  **ICD-10-CA:**  E102, E112, E132, E142, I12, I13, N08, N18, N19 |
| Liver Disease | OHIP, DAD, NACRS | **OHIP Dxcode:**  571  **ICD-10-CA:**  K70 K71 K72 K73 K74 K75 K76 K77 |
| Primary Care Visits | OHIP | **OHIP Feecode:**  A001, A002, A003, A007, A903, E075, G212, G271, G372, G373, G365, G538, G539, G590, G591, K005, K013, K017, P004, K130, K131, K132, K030 |

#
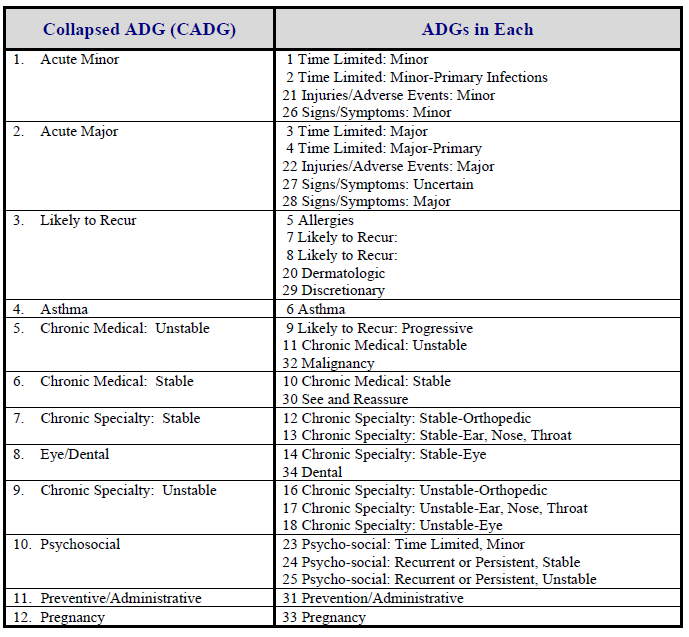
Appendix B: John’s Hopkins Aggregated Diagnosis Groups

# Appendix C: Abbreviations

ACG – Johns Hopkins Adjusted Clinical Group system

ADG – Johns Hopkins Aggregated Diagnosis Groups

ALR – Cancer Activity Level Reporting database

CAPE – Client Agency Program Enrolment database

CCM – Comprehensive Care Model

CHC – community health centre

CIHI-DAD or DAD – Canadian Institute for Health Information Discharge Abstract Database

CIHI-NACRS or NACRS – Canadian Institute for Health Information National Ambulatory Care Reporting System

COPD – chronic obstructive pulmonary disease

DCLASS – drug class

DIN – drug identification number

DOLC – Date of last health care contact

ED – emergency department

EXCL – opioids to exclude

FHG – Family Health Group

FHN – Family Health Network

FHO – Family Health Organization

GEE – generalized estimating equation

ICD-10-CA – Tenth Revision of the International Statistical Classification of Diseases and Related Health Problems, Canadian modification

IKN – ICES number (unique identifier)

IPDB – ICES Physician Database

IQR – interquartile range

LA – long-acting

LHIN – Local Health Integration Network

LTC – long-term care

MAID – opioids used for medical assistance in dying

MHA – Mental Health and Addictions

NMS – Narcotics Monitoring System

OAT/OMT – opioid agonist therapy

ODPRN – Ontario Drug Policy Research Network

OHIP – Ontario Health Insurance Plan

OMHRS – Ontario Mental Health Reporting System

RPDB – Registered Persons Database

RUNS – opioids used for the treatment of diarrhea

SD – standard deviation

SDS – Same Day Surgery

THC – tetrahydrocannabinol
