# Supplementary material for: Inequities in access to primary care among opioid recipients in Ontario, Canada: A population-based cohort study
Source: PLoS Med. 2021 Jun 1;18(6):e1003631. doi: 10.1371/journal.pmed.1003631 (PMC8168863; doi:10.1371/journal.pmed.1003631)
Supplement: Attachment — Submitted filename: gomes.pdf [file pmed.1003631.s007.pdf]

This paper studies access to primary care in Ontario and its relationship to opioids.

I was asked for a statistical report and I interpret that to include all aspects of the design and conduct of the study. The version I received did not have page numbers. My numbering has the abstract starting on page 2

Points of detail

Page 4 Is the figure of 1 in 6 typical world–wide or is this specific to North America?

Page 9 To be picky the authors used quintile categories, of which there are five, not quintiles of which there are four.

Page 9 Testing the proportional hazards assumption is a good idea but reporting it would be even better and I cannot find that. Showing us the plots would also be a worthwhile addition.

Page 11 As far as I can see this is the first mention of a capitation–based model. and it is not really explained here. I did struggle with some of the description as the Ontario system is not as familiar to me as it obviously is to the authors. Some more background would be helpful.

Page 11 While it is true that the OPT participants did not differ from the unexposed after adjustment it is clear from Table 2 that adjustment had a substantial effect which I would have thought merited some explanation and further comment. Adjustment also does not seem to have brought about a narrowing of the confidence intervals which one might have been expecting.

Page 12, Table 3 Why has the approach to analysis been changed here? Before the main interest was in comparing the three groups but now we have separate before and after comparisons.

Table 1 I am not sure having a table which spans so many pages is optimum for reader accessibility. Despite the caption I did not spot any censored values here.

Table S2 Does this mean that OAT participants all got their most recent prescription outside the primary care setting (≤ 5)? If so where did they obtain it?

Table S2 If the number of prescribers for OPT participants is important then I think an IQR of 1 to 1 is rather uninformative and I might give

the actual frequencies. Even just the number with 1 or more than 1 might be better. I assume it cannot be zero.

Table S4 I assume ≤ 5 is a typo for **?

Summary

This is a detailed account of the analysis of these data–sets and my comments are mostly for clarification. I do not have major concerns about the analysis strategy.

Michael Dewey
