# Supplementary material for: Inequities in access to primary care among opioid recipients in Ontario, Canada: A population-based cohort study
Source: PLoS Med. 2021 Jun 1;18(6):e1003631. doi: 10.1371/journal.pmed.1003631 (PMC8168863; doi:10.1371/journal.pmed.1003631)
Supplement: Attachment — Submitted filename: Response to Reviewer Comments - Feb 3 2021.docx [file pmed.1003631.s008.docx]

**COMMENTS FROM ACADEMIC EDITOR:**

**1. I think this paper is generally well-done, and I think the fact that 4 reviewers have endorsed a minor revision is a meaningful metric in its favor.**
We thank the editor and reviewers for their review and supportive comments.  **2. However, there are a couple of concerns that I think deserve emphasis. First, the authors should really try to make clear why the PCP was lost. Was it through patients walking away from their PCPs or loss of follow-up, or was it through the PCPs letting the patient go, moving, or some other physician-side cause. Whether the relationship was lost due to patient-side or physician-side explanations would be expected to play a role in determining reconnections to primary care.**
We thank the editor for raising this important point. In Table 1, we include the reasons for patients being de-rostered from their PCP. The majority in all groups were due to the physician terminating the patient enrolment (88.8% among OAT recipients, 78.1% among long-term OPT recipients, and 79.5% among the rest of the population). We have now included these findings in the text of the manuscript and have conducted a sensitivity analysis among those individuals where the physician terminated the enrolment. The findings of this sensitivity analysis were consistent with the main findings.

**Inserted Text**

**Methods:** *In two post-hoc sensitivity analyses, we refit models for the primary outcome among individuals whose physician ended the patient enrolment, and stratified by OAT type.*

**Results:
a)** *The majority of people in each of the patient groups had their enrolment terminated by their physician, with a higher percentage of OAT recipients losing enrolment for this reason (88.8%) compared to long-term OPT recipients (78.1%) and unexposed individuals (79.5%; Table 1).*

**b)** *In our sensitivity analysis restricted to individuals whose physician ended the patient enrolment, the results were consistent with the primary analysis.*

**Excerpt from Table 2**

| **Opioid exposure** | **Individuals** | **N (rate per 1,000 person-days)** | **Unadjusted Hazard Ratio (95% CI)** | **Adjusted Hazard Ratio (95% CI)** |
| --- | --- | --- | --- | --- |
| **Sensitivity analysis: Primary outcome among those whose physician ended enrolment** | | | | |
| **Opioid unexposed individuals** | 118,861 | 45,641 (1.46) | 1 | 1 |
| **OAT recipient** | 1,533 | 358 (0.79) | 0.55 (0.50-0.61) | **0.53 (0.47-0.58)** |
| **Long-term OPT recipient** | 2,845 | 1,477 (2.44) | 1.60 (1.52-1.69) | **0.96 (0.91-1.02)** |

**3. Second, the generalizability issue is important, because one province in Canada may have provincial policies or regional explanations for these results that don't apply elsewhere.**

We appreciate the editor and reviewer for their suggestions regarding better contextualization of the generalizability of our findings elsewhere in Canada and the United States. We have now expanded our discussion to include more details on the generalizability of this work and believe that it greatly improves the manuscript.

**Inserted Text (Discussion):**
*The generalizability of our findings elsewhere in Canada, and across North America, and potential policy implications warrant further discussion. For the last two decades, Canada has been undergoing considerable primary health care reform, with a focus on equitable access to care, integration of care and team-based primary care models.[31, 32] Although there has been inter-provincial variation in the pace and extent of the reforms [31], the findings from our study are likely generalizable across the country. In contrast, because the healthcare system in the United States is funded by a mix of public and private insurers, access to primary care for people with complex needs or low socioeconomic status differs from that in Canada.[33, 34] This could impact the applicability of our findings to the U.S. population; however it is likely that barriers to accessing primary care are larger outside of a universal publicly-funded healthcare system, which could heighten the disparities identified among people with an OUD. Therefore, although the structure of primary care differs across North America, our findings suggest that even in a province with a publicly-funded healthcare system that has undergone considerable primary care transformation, barriers to care continue to exist for people who use opioids, particularly those with an OUD. Future efforts are needed to more fully understand the patient and provider-level factors that contribute to these gaps, including financial disincentives within reimbursement models, addressing stigma and discrimination within the healthcare system, and creating safe environments for marginalized populations.*
 **4. Third, not all opioid use and opioid antagonist use is alike. The duration, intensity, and type of use should be considered when categorizing subjects into the groups here.**
We agree with the editor, which is why we restricted our cohort of opioid recipients to people with evidence of long-term opioid use using criteria that align with guideline definitions of chronic opioid use (90 days of ongoing use). We believe that this helps ensure that we are focused on a population with ongoing, long-term opioid needs. We have further described the characteristics of opioid use in this group in our supplementary appendix (Table S2) which shows a median daily dose of 45MME. To address the question regarding OAT duration and intensity, we have expanded Table S2 to include details on the number of days of treatment with OAT over the past month, and have conducted a sensitivity analysis where we stratified our primary model according to OAT type.
 **Inserted Text:
Methods:** *Finally, in two post-hoc sensitivity analyses, we refit models for the primary outcome among individuals whose physician ended the patient enrolment, and stratified by OAT type.*

**Results:** *Similarly, the results of the primary analysis were consistent between both OAT types (Table 2).*

| **Opioid exposure** | **Individuals** | **N (rate per 1,000 person-days)** | **Unadjusted Hazard Ratio (95% CI)** | **Adjusted Hazard Ratio (95% CI)** |
| --- | --- | --- | --- | --- |
| **Sensitivity analysis: Primary outcome, stratified by OAT type** | | | | |
| **Opioid unexposed individuals** | 149,599 | 63,232 (1.68) | 1 | 1 |
| **Methadone recipient** | 1,319 | 321 (0.82) | 0.50 (0.45-0.56) | **0.52 (0.46-0.58)** |
| **Buprenorphine/naloxone recipient** | 404 | 128 (1.15) | 0.70 (0.58-0.83) | **0.66 (0.56-0.79)** |
| **Long-term OPT recipient** | 3,644 | 2,009 (2.69) | 1.53 (1.47-1.60) | **0.96 (0.92-1.01)** |

**REQUESTS FROM INTERNAL EDITORS:

1. Please structure your abstract using the PLOS Medicine headings (Background, Methods and Findings, Conclusions). In the last sentence of the Abstract Methods and Findings section, please include a note about any key limitation(s) of the study's methodology.**
This change has been made.  **Inserted Text (Abstract):**
*The main limitation to our findings relates to their generalizability outside of primary care enrolment models and in jurisdictions with different financial incentives incorporated into primary care provision.* **2. In the abstract (methods and findings subsection), we'd suggest noting briefly the type of analysis conducted (multivariable analysis) and the confounders adjusted for.**

This change has been made.

**Edited Text (Abstract):**
*We fit multivariable models assessing the primary outcome of primary care re-attachment within one year, adjusting for demographic characteristics, clinical comorbidities, and health services utilization. Secondary outcomes included rate of emergency department (ED) visits and opioid toxicity events.* **3. At this stage, we ask that you include a short, non-technical Author Summary of your research to make findings accessible to a wide audience that includes both scientists and non-scientists. The Author Summary should immediately follow the Abstract in your revised manuscript. This text is subject to editorial change and should be distinct from the scientific abstract. Please see our author guidelines for more information:** [**https://journals.plos.org/plosmedicine/s/revising-your-manuscript#loc-author-summary**](https://journals.plos.org/plosmedicine/s/revising-your-manuscript#loc-author-summary)
We have now included an Author Summary directly below the Abstract in our revised manuscript. **4. Please reformat the citation style into PLOS Medicine's format (should be straight forward if using referencing software) - this should use callouts formatted as sequential numerals in square brackets (not superscript).**
This change has been made. **5. Please clarify in the paper if the analytical approach followed here corresponded to one laid out in a prospective protocol or analysis plan? Please state this (either way) early in the Methods section.**

**a) If a prospective analysis plan (from your funding proposal, IRB or other ethics committee submission, study protocol, or other planning document written before analyzing the data) was used in designing the study, please include the relevant prospectively written document with your revised manuscript as a Supporting Information file to be published alongside your study, and cite it in the Methods section. A legend for this file should be included at the end of your manuscript.

b) If no such document exists, please make sure that the Methods section transparently describes when analyses were planned, and when/why any data-driven changes to analyses took place.  

c) In either case, changes in the analysis-- including those made in response to peer review comments-- should be identified as such in the Methods section of the paper, with rationale.**

The analytical approach followed a prospective analytic plan which is now provided as a Supporting Information file. This has been cited in the Methods section.

**Inserted Text (Methods):**
*The study design and analyses were described in a prospective analytic plan (Supplementary File). Analyses were conducted at ICES using SAS Enterprise Guide, version 7.1 (SAS Institute, Inc. Cary, NC) and used a type 1 error rate of 0.05 as the threshold for statistical significance.*

**6. We'd suggest the authors use an appropriate reporting guideline pertaining to the study type to support more detailed reporting of the methods and findings; since the study involved routinely-collected clinical data and linkage of datasets, the most appropriate might be the RECORD guideline (**[**https://www.equator-network.org/reporting-guidelines/record/**](https://www.equator-network.org/reporting-guidelines/record/)**). If the authors use this, please ensure that the study is reported according to the RECORD guideline, and include the completed  checklist as Supporting Information.  Please add the following statement, or similar, to the Methods: "This study is reported as per the RECORD guideline (S1 Checklist)". When completing the checklist, please use section and paragraph numbers, rather than page numbers.**
We thank the editors for making this suggestion and have now provided the RECORD checklist in the Supporting Information.  **7. In the discussion section, it's not clear that currently the authors acknowledge the possibility for bias due to confounding by unmeasured factors in the study, ie that rather than opioid use resulting in the failure to re-enrol with a PCP but rather some other underlying factor is linked with increased risk of opioid use and with failure to re-enrol with a PCP (eg, socioeconomic factors could be plausibly imagined here). This could be better discussed.**
We thank the editors for flagging this. We have now incorporated a discussion of unmeasured confounding and its potential impacts, particularly as it related to the cohort of long-term OPT recipients.

**Inserted Text (Discussion, Limitations section):**

*Finally, as is the case with observational research generally, it is possible that unmeasured confounding impacted some of our findings. In particular, unadjusted models indicated that long-term OPT recipients were generally more likely to secure a primary care provider within the year, but this association disappeared after adjustment. This likely reflects the high comorbidity burden observed among chronic pain patients in this study which may lead them to more quickly seek a new primary care provider.*

**COMMENTS FROM REVIEWERS

Reviewer #1: Michael Dewey**

**1. Page 4 Is the figure of 1 in 6 typical world-wide or is this specific to North America?**
We can confirm that this figure was obtained from studies conducted in the US and Canada. While inequities of access to primary care have been studied elsewhere (primarily in the United Kingdom and US[1]), statistics such as the one provided in the background are not easily obtained outside of North America, and given the focus of our study population, we felt it best to provide context for access to primary care in North American populations.

**2. Page 9 To be picky the authors used quintile categories, of which there are five, not quintiles of which there are four.**
We have used terminology that is consistent with prior research reporting income quintiles (when there are 5 categories). We believe that the quintiles as currently reported are accurate, and have therefore not made any changes to avoid confusing the reader. We would be happy to reconsider if the editors would prefer this to be clarified in the text.

**3. Page 9 Testing the proportional hazards assumption is a good idea but reporting it would be even better and I cannot find that. Showing us the plots would also be a worthwhile addition.**
We thank the reviewer for suggesting this, and have now included details of the tests of proportional hazards in the Supplemental Appendix (Figure S1)

**4. Page 11 As far as I can see this is the first mention of a capitation-based model. and it is not really explained here. I did struggle with some of the description as the Ontario system is not as familiar to me as it obviously is to the authors. Some more background would be helpful.**
We thank the reviewer for making this suggestion, which was also made by other reviewers who are unfamiliar with the Ontario system. We have now incorporated a paragraph providing more detail on the Ontario primary care system in the background of the manuscript.

**Inserted Text (Introduction):**
*In Ontario, primary care is predominantly provided through primary care enrolment models (PEMs) that include capitation models (physicians are primarily paid through age- and sex-based reimbursements per patient), enhanced fee-for-service models (physicians are mainly paid through fee-for-service billings), and other specialized models where care is targeted to specific populations.[20, 21] Alternatively, primary care can be delivered through community health centres (CHCs), which are comprised of multidisciplinary teams that aim to serve populations who may experience issues accessing health services, or through traditional fee-for-service arrangements.[20] Under PEMs, physicians provide comprehensive primary care to enrolled patients, and are prohibited from refusing enrolment due to patient health status or high service need.[20] However, despite this, qualitative studies among opioid recipients cite ongoing challenges securing primary care, which may be influenced by stigma and financial disincentives for complex patients within some PEMs.[11]*

**5. Page 11 While it is true that the OPT participants did not differ from the unexposed after adjustment it is clear from Table 2 that adjustment had a substantial effect which I would have thought merited some explanation and further comment. Adjustment also does not seem to have brought about a narrowing of the confidence intervals which one might have been expecting.**
We thank the reviewer for pointing out this omission. We have now included a brief discussion of this in our revised manuscript. However, because the confidence intervals in fact did narrow after adjustment, we have not specifically mentioned this, and have instead focused on the large change in hazard ratios after adjustment (which is likely due to the high comorbidity burden of chronic pain patients receiving long-term OPT).

**Inserted Text (Discussion - limitations)**:
*Finally, as is the case with observational research generally, it is possible that unmeasured confounding impacted some of our findings. In particular, unadjusted models indicated that long-term OPT recipients were generally more likely to secure a primary care provider within the year, but this association disappeared after adjustment. This likely reflects the high comorbidity burden observed among chronic pain patients in this study which may lead them to more quickly seek a new primary care provider.*

**6. Page 12, Table 3 Why has the approach to analysis been changed here? Before the main interest was in comparing the three groups but now we have separate before and after comparisons.**
We thank the reviewer for raising this question. We changed our analytic approach for this outcome because we knew that risks of opioid toxicity and emergency department visits are intrinsically different between groups, regardless of primary care provider. Therefore, to compare these outcomes after primary care provider loss would likely show higher rates among long-term OPT and OAT cohorts when this didn’t reflect a change due to provider loss. Therefore, for this analysis we decided that the most appropriate comparison would be one that evaluated whether risk of these outcomes increased during the period of provider loss compared to the baseline rates for that opioid therapy group prior to losing their primary care provider.

**Inserted Text (Methods):**
*We compared rates of our secondary and tertiary outcomes during follow-up to rates in the 1 year prior to termination of enrolment because these rates already differed considerably between opioid therapy groups at baseline, and opioid unexposed individuals were required to have had no opioid toxicity event in the prior 3 years. Therefore, within person analyses of rates before and after primary care provider loss were determined to be the most appropriate comparison.*

**7. Table 1 I am not sure having a table which spans so many pages is optimum for reader accessibility. Despite the caption I did not spot any censored values here.**
We have now split table 1 into two tables which present the demographic characteristics (Table 1a) and clinical characteristics and health services utilization (Table 1b) of the cohort. We believe that this will help maintain all relevant cohort information while ensuring that the individual tables aren’t too long. We have removed the caption regarding censored values as the reviewer is correct and there are none in these tables.

**8. Table S2 Does this mean that OAT participants all got their most recent prescription outside the primary care setting (≤ 5)? If so where did they obtain it?**
Yes – this is correct. Across North America, the majority of methadone is provided through specialized clinics, and overseen by addictions medicine physicians. In Ontario, buprenorphine/naloxone is provided more frequently through primary care; however, because of the commitment of these primary care physicians to providing substance use disorder treatment, it is unlikely that they would de-roster their patients; and therefore it is unlikely that these patients would end up in our study cohort. Because of this, it is entirely expected that the vast majority of patients with OAT who lose their primary care providers would be accessing their OAT elsewhere. However, it is important to note that clinics specializing in OAT (e.g. methadone clinics) do not provide primary care services, and so – despite having access to OAT elsewhere, these patients would no longer have access to primary care services.
 **9. Table S2 If the number of prescribers for OPT participants is important then I think an IQR of 1 to 1 is rather uninformative and I might give the actual frequencies. Even just the number with 1 or more than 1 might be better. I assume it cannot be zero.**
We thank the reviewer for making this suggestion. This change has been made (Table S2)

**10. Table S4 I assume 5 is a typo for **?**
We thank the reviewer for flagging this question. We have now revised Table S4 for clarity.

**11. Summary: This is a detailed account of the analysis of these datasets and my comments are mostly for clarification. I do not have major concerns about the analysis strategy.**
We thank the reviewer for this supportive comment and hope that our changes above have addressed their questions.

**Reviewer #2:

1. This manuscript focuses on differential access to primary care between three groups of people: 1) those receiving opioid agonist therapy (OAT); 2) those receiving long-term opioid pain treatment (OPT); 3) and those not exposed to opioids to primary care providers.  Using a retrospective cohort study design, the authors examine data of individuals from Ontario, Canada, whose enrolment with a physician practicing primary care (PCP) was terminated between January 2016 and December 2017.  The application of eligibility criteria resulted in a cohort of 154,970 Ontarians who lost their PCP during the relevant timeframe.  The primary outcome was primary care re-attachment within one year.  Secondary outcomes included rate of emergency department visits and opioid toxicity events.   
Overall, the manuscript focuses on an important area of inequities in access to primary care.  The focus on individuals receiving opioids for long-term chronic pain and to treat Opioid Use Disorder is timely given the attention to the dearth of treatments for non-cancer pain and the overprescribing of opioids that has resulted in an epidemic of opioid misuse, overdose deaths, and Opioid Use Disorder.  The key strength of the study is the use of a large cohort of linked- patient level data providing the ability to look at loss of primary care, re-engagement in primary care, and the utilization of health care services when primary care was not available.

 The main weakness of the study (aside from those the authors list in the discussion) is potential lack of generalizability across other provinces in Canada or other parts of the world devastated by the opioid crisis like the United States.  The significance of the research could be improved substantively if the authors placed their results within models of healthcare delivery for the rest of Canada and other parts of North America (especially the United States) and offered potential policy changes to address differential access as well as future areas of research.  

As stated above in the summary and overall impressions, the significance of this research is limited by generalizability to a single province in Canada.  While the data did come from Ontario, the authors could compare primary care models from this single province to other parts of Canada and the United States and the potential implication of these findings.  As written, the authors do not even discuss potential primary care practice, policy, or research implications of their findings under the limited application to Ontario.  It seems there is an opportunity to lay out these implications within Ontario and apply them to an understanding of primary care in other portions of North America.**

We thank the reviewer for highlighting this omission. We have now expanded our discussion of the implications of our findings, and have spoken more to the generalizability of these findings to elsewhere in Canada and the United States. We believe that this has greatly strengthened the discussion of our findings and thank the reviewer for making this suggestion.

**Inserted Text (Discussion):**
*The generalizability of our findings elsewhere in Canada, and across North America, and potential policy implications warrant further discussion. For the last two decades, Canada has been undergoing considerable primary health care reform, with a focus on equitable access to care, integration of care and team-based primary care models.[31, 32] Although there has been inter-provincial variation in the pace and extent of the reforms [31], the findings from our study are likely generalizable across the country. In contrast, because the healthcare system in the United States is funded by a mix of public and private insurers, access to primary care for people with complex needs or low socioeconomic status differs from that in Canada.[33, 34] This could impact the applicability of our findings to the U.S. population; however it is likely that barriers to accessing primary care are larger outside of a universal publicly-funded healthcare system, which could heighten the disparities identified among people with an OUD. Therefore, although the structure of primary care differs across North America, our findings suggest that even in a province with a publicly-funded healthcare system that has undergone considerable primary care transformation, barriers to care continue to exist for people who use opioids, particularly those with an OUD. Future efforts are needed to more fully understand the patient and provider-level factors that contribute to these gaps, including financial disincentives within reimbursement models, addressing stigma and discrimination within the healthcare system, and creating safe environments for marginalized populations.* **Minor Issues:
2. The authors do not lay out the different models for providing primary care until the discussion.  It would be helpful to orient the reader to these different models earlier in the paper, perhaps even in the introduction.  Including a table with the different models could be useful and referenced in the appropriate places throughout the paper.**  
We thank the reviewer for making this suggestion – we have now expanded our description of the primary care models, and have moved this up from the discussion to the introduction of the paper. We have also cited a paper that has a helpful table comparing the different models of primary care. We have not recreated this table in our manuscript because we feel that we have provided the relevant information in the text and that readers are directed to the prior publication which provides a strong foundation in primary care models in Ontario (citation [2] in the reference list in this document).

**Inserted and Revised Text (Introduction):**
*In Ontario, primary care is predominantly provided through primary care enrolment models (PEMs) that include capitation models (physicians are primarily paid through age- and sex-based reimbursements per patient), enhanced fee-for-service models (physicians are mainly paid through fee-for-service billings), and other specialized models where care is targeted to specific populations.[20, 21] Alternatively, primary care can be delivered through community health centres (CHCs), which are comprised of multidisciplinary teams that aim to serve populations who may experience issues accessing health services, or traditional fee-for-service arrangements.[20] Under PEMs, physicians provide comprehensive primary care to enrolled patients, and are prohibited from refusing enrolment due to patient health status or high service need.[20] However, despite this, qualitative studies among opioid recipients cite ongoing challenges securing primary care, which may be influenced by stigma and financial disincentives for complex patients within some PEMs.[11]*

**3. In the current climate, I as a reader misinterpreted the term "inequities" in access in the title.  I was prepared to see at least some data presented by race/ethnicity to look at inequities in access to primary care.  People have intersecting identities that lead to inequitable access to healthcare including the receipt of opioids but also the combination of receiving opioids with race/ethnicity.  As a population- based study, these data probably exist and in addition to age and gender could be important to examine.  If there is a lack of diversity, it warrants a mention and impacts generalizability of findings.**
We thank the reviewer for highlighting this potential interpretation of the phrase ‘inequities’. We unfortunately do not have ethnicity/race data within the data repository at ICES, but used the Ontario Marginalization Index’s Ethnic Concentration measure as a way of capturing ethnocultural diversity based on neighbourhood characteristics. These data are presented in Table 1a in the revised manuscript. Although there were some small differences in distribution of OAT recipients and long-term OPT recipients as it relates to ethnic concentration in place of residence, these differences were not particularly consistent and therefore – although we did adjust for this measure in our models – we did not find it appropriate to speak to these findings specifically in the manuscript. We have not removed the word ‘inequities’ from our title as we believe that as phrased it is clear that this represents inequity in access to primary care according to opioid use. However, if the editors disagree, we would be happy consider alternative language.

**4. Sentence 3 of the introduction is a bit confusing and could be improved by either re-writing the sentence or breaking into two sentences.**
We have revised the sentence for clarity.

**Revised text (Introduction, 3^rd^ sentence):**
*Importantly, those without access to primary care often have lower socioeconomic status and fewer social and community supports.[6, 7]*

**5. Stigma is referenced in the abstract and conclusions but no where else in the paper.  There is an extensive literature on stigma toward people using opioids to treat chronic pain and OUD.  In addition, there are papers that talk about stigma toward providers and medications used to treat OUD.  The introduction would be strengthened to include some reference to this literature.  Likewise, if stigma is to be mentioned in a sentence of the conclusions some link to the study findings is important.  This important area could be considered more thoughtfully with additional mention in the introduction and implications of study findings.  When stating "ongoing efforts are needed to address the stigma and discrimination that may introduce barriers to the healthcare system" it would be helpful to state more clearly how study results support this statement and exactly what efforts might be helpful.**
We thank the reviewer for raising this question as we believe that speaking to the literature on stigma and discrimination for people who use drugs and those with chronic pain is important. We mention this in the introduction, paragraph 2, where we discuss some of the literature around stigma for patients and physicians (see excerpt below). We have also expanded our discussion of this as it relates to primary care enrolment models in a new 3^rd^ paragraph of the introduction. Finally, we speak to stigma in the 3^rd^ paragraph of our discussion. We believe that the concept of stigma and how it relates to this study is integrated throughout the manuscript.

**Excerpt (Introduction paragraph 2):**
*However, recent research has demonstrated that achieving this quality of care can be particularly challenging for these patients who may be labeled as ‘undesirable’ to physicians due to stigma, high health care needs, or physicians’ lack of comfort prescribing opioids and concerns of adverse events.[6, 11-13] Surveys conducted among physicians reinforce this, showing that up to 40% of primary care physicians would not accept a patient who required opioid treatment into their practice.[12, 14, 15] There is particular concern that this has been exacerbated in recent years with the publication of clinical guidelines for opioid prescribing in chronic non-cancer pain[16, 17] that recommended changes in opioid prescribing patterns across North America, sometimes straining the patient-physician relationship.[11, 18, 19]*

**Inserted Text (Introduction, paragraph 3):**
*Under these PEMs, physicians provide comprehensive primary care to enrolled patients, and are prohibited from refusing enrolment due to patient health status or high service need.[20] However, despite this, qualitative studies among opioid recipients cite ongoing challenges securing primary care, which may be influenced by stigma and financial disincentives for complex patients within some PEMs.[11]*

**6. Introduction section provides references about physicians' discomfort or unwillingness to treat patients receiving opioids but neglect to include financial incentives/disincentives.   It is important to add this to the introduction since finances are an important contributor to primary care providers not wanting to treat complex patients receiving opioids.**
We agree and have now moved up our discussion of the primary care models in Ontario and potential financial disincentives to the introduction.

**Inserted Text (Introduction):**
*Under these PEMs, physicians provide comprehensive primary care to enrolled patients, and are prohibited from refusing enrolment due to patient health status or high service need.[20] However, despite this, qualitative studies among opioid recipients cite ongoing challenges securing primary care, which may be influenced by stigma and financial disincentives for complex patients within some PEMs.[11]*

**7. Last sentence of the introduction states: "Therefore, we conducted a large, population-based cohort study comparing time to securing a new primary care physician among people with varying histories of opioid use who had recently lost their primary care provider."   Data were analyzed and reported based on whether someone was or was not reenrolled in primary care.  Data were not analyzed or reported based on "time to securing a primary care physician" although if patient level data are available based on dates of initial re-enrollment that would be an additional interesting way to report on results and is strongly encouraged.  If data are not available, rewriting this sentence to more clearly recommend study findings is recommended.**  
We used this language because we undertook a time-to-event analysis where we censored individuals over follow-up and incorporated time-to-securing primary care provider into our statistical model. However, we understand how this could be confusing as we don’t report the time to securing the provider in our analysis. We have therefore simplified our objective statement as suggested by the reviewer.

**Edited Text (last paragraph of introduction):**
*Therefore, we conducted a large, population-based cohort study comparing likelihood of securing a new primary care physician among people with varying histories of opioid use who had recently lost their primary care provider.*

**8. Under Data Sources, first sentence: spell out ICES the first time it appears in the paper.**    
In 2018, the institute formerly known as the Institute for Clinical Evaluative Sciences formally adopted the initialism ICES as its official name. This change acknowledges the growth and evolution of the organization’s research since its inception in 1992, while retaining the familiarity of the former acronym within the scientific community and beyond. Therefore, although ICES is no longer considered an acronym, we have explained the previous meaning of ICES in the revised manuscript.

**Revised text (Methods):**
*We obtained data from ICES (formerly known as the Institute for Clinical Evaluative Sciences), an independent, non-profit research institute whose legal status under Ontario’s health information privacy law allows for the collection and analysis of health care and demographic data.*
 **9. I could not find the citation for reference 20, although I may have overlooked it.**
Reference 20 refers to a paper by Hutchinson et al and describes primary care models in Ontario. We have confirmed that it is cited frequently in the introduction in the manuscript. **Reviewer #3:

This paper examines primary care access among people on long-term opioid analgesic prescriptions and opioid agonist therapies. The paper is very well-written and covers an important and timely topic of inequities in a highly vulnerable group. Comments for the authors' consideration are listed below.

1.    The method used to identify the OAT and OPT recipients seems to risk missing people with opioid use disorder who are not on agonist treatments. For example, someone who is obtaining opioids illegally but not receiving OAT. This limitation is acknowledged, but if you have room it would be beneficial to see a bit more on what the implications are for the study results.**
As the reviewer mentions, this was listed as a limitation because we are unable to capture opioid use disorder (OUD) that does not lead to treatment with OAT in our data. However, we agree that we can discuss these implications more clearly. We have expanded our discussion of this subject in our revised manuscript.

**Inserted Text (Discussion):**
Third, we were unable to identify people with an OUD who were not receiving OAT. Therefore, we are unable to determine whether their patterns of primary care access differ from those receiving treatment for OUD. However, based on the literature regarding negative physician perceptions of people who use drugs[9, 11, 15], we anticipate that the findings among OAT recipients would be similar for those with OUD who are not actively receiving treatment. **2.    Given the variability across countries in opioid agonist prescribing policies, can you add a note about whether there are any restrictions on agonist prescribing that might confound the results? For example, is there any additional training to prescribe these medications, restrictions on the settings in which they can be provided or caps on the number of prescriptions that can be written?**
In Ontario, there are currently no restrictions on who can prescribe OAT, although at the time of conducting this study, methadone prescribers required a Health Canada exemption (buprenorphine/naloxone prescribers did not). This has led to buprenorphine/naloxone being more broadly integrated into primary care, while methadone has largely been provided through independent methadone clinics. In our study, we found that very few (<=5) individuals who lost their primary care provider in the OAT cohort were actively receiving their OAT treatment from that physician (Table S2 in supplemental appendix), suggesting that they were receiving their treatment for OUD elsewhere. While this is likely largely driven by the fact that most people access OAT outside of their primary care provider, it also highlights the fact that those who are receiving OAT from their primary care provider are very unlikely to have lost that provider (hence they are not included in this study). This is likely reflective of the benefits of integration of OAT into primary care whenever possible. We have now included a paragraph describing these findings in the discussion section of our manuscript.

**Inserted text (Discussion):**
*Another important finding is that it was also extremely rare that primary care providers were the physician actively prescribing OAT to their patients at time of enrolment loss. Although over the study period, physicians in Canada required federal approval before prescribing methadone, this is no longer the case, and has never been a requirement to prescribe buprenorphine/naloxone.[27] Therefore, the fact that nearly all OAT recipients who had lost their primary care provider in our study were receiving treatment for OUD outside of their primary care provider is suggestive of fragmentation in the healthcare system for these patients. Given the considerable economic and clinical benefits of integration of OAT into primary care[28], concerted effort to incentivize this integration throughout Canada is needed.*

**3.    Although I agree with the authors that the most likely explanation is due to problems on the provider and systems end (e.g., stigma, logistical barriers in accessing care), this cannot be assumed given this design. For example, "Also concerning is our finding that the rate of ED visits increased significantly during follow-up for long-term OPT recipients, suggesting that their loss of a primary care provider led them to seek care in a hospital setting." Consider adding some text describing alternative possibilities here. It seems that this could also reflect a worsening course in this cohort - people were starting to struggle, discontinued treatment and understandably needed more intensive care in that context. This interpretation might lead to different strategies to support this group (e.g., how do we re-engage people in care or implement other low-barrier access to services). More balance in the discussion about the possible reasons for this would strengthen the paper.**
We thank the reviewer for this suggestion, and we have now revised our discussion section to speak to the potential of worsening clinical comorbidities leading to higher ED visits.  **Revised Text (Discussion):**
*Also concerning is our finding that the rate of ED visits increased significantly during follow-up for long-term OPT recipients, suggesting that their loss of a primary care provider led them to seek care in a hospital setting. While we also observed a significant increase among opioid-unexposed individuals, the rate of ED visits and the increase during follow-up was much higher among long-term OPT recipients, which may also be reflective of worsening clinical comorbidities among chronic pain patients who lose access to their primary care provider. Interestingly, among OAT recipients, we observed no significant change in the rate of ED visits.*

**Reviewer #4:

This retrospective cohort study examines a primary outcome of re-attachment to primary care for individuals across three groups - long-term opioid pain therapy (OPT), opioid agonist therapy (OAT), or no opioids. The overall aim was examining differences in care for those with OUD, prescribed opioids for pain, and no opioids. Findings indicated that those being treated for OUD were less likely to find PCPs within 1 year. Strengths of this manuscript are examining a large retrospective sample, use of advanced statistical approaches, and targeting an important topic -disparities in access to primary healthcare for those with opioid use and chronic pain.

There are a few things that would benefit from clarification and elaboration in a revision of this manuscript.

1. Abstract: Suggest changing spelling: enrolment to enrollment (though this may be country-specific preference).**
We thank the reviewer for identifying this inconsistency. We have consistently used enrolment throughout the text as this is the Canadian spelling of this term, but we would be happy to change if preferred by the editors. **2. Introduction - well written. It may be helpful for some description of healthcare in Ontario and elsewhere in Ontario, for those unfamiliar with this healthcare system or primary care enrollment models (PEM). For instance, is enrollment in these programs mandatory?**
We thank the reviewer for identifying this gap, which was also mentioned by other reviewers. We have now expanded our description of the PEMs in the introduction.

**Inserted Text (Introduction):**
*In Ontario, Canada, primary care is predominantly provided through primary care enrolment models (PEMs) that include capitation models (physicians are primarily paid through age- and sex-based reimbursements per patient), enhanced fee-for-service models (physicians are mainly paid through fee-for-service billings), and other specialized models where care is targeted to specific populations.[20, 21] Alternatively, primary care can be delivered through community health centres (CHCs), which are comprised of multidisciplinary teams that aim to serve populations who may experience issues accessing health services, or through traditional fee-for-service arrangements.[20] Under PEMs, physicians provide comprehensive primary care to enrolled patients, and are prohibited from refusing enrolment due to patient health status or high service need.[20] However, despite this, qualitative studies among opioid recipients cite ongoing challenges securing primary care, which may be influenced by stigma and financial disincentives for complex patients within some PEMs.[11]*
 **3. Methods - What is rationale for removing individuals receiving palliative care for chronic pain? Palliative care does not always mean "end of life" and therefore many patients receiving palliative care may fall into the category of long-term opioid pain therapy (OPT) in this study. Suggest providing rationale - not necessarily changing exclusion criteria.**
We made this exclusion because a patient’s move to palliative care could influence their source of primary care and health services utilization. We have included mention of this in the revised manuscript.

**Revised text (Methods – Identification of Cohort):**
*We excluded people younger than 18 or older than 105 on the date enrolment changed, those without health care contact in the 8 years before the enrolment status change, and people who resided out of province or were not eligible for OHIP on the status change date. We excluded recipients of palliative care services in the preceding year since this may influence primary care needs and follow-up, and excluded those enrolled with their rostering physician for less than one year prior to the status change date to ensure we were studying individuals with an ongoing patient-provider relationship.*
 **4. Were individuals prescribed Naltrexone included? If not, why? (provide rationale).**
We did not include naltrexone in our definition of OAT in this study because the vast majority of naltrexone use in Ontario is for alcohol use disorder.

**Inserted Text (Methods – Exposure):**
*We did not include naltrexone in the inclusion criteria for OAT recipients because it is predominantly used to treat alcohol use disorder in Ontario.* **5. Why are buprenorphine and naloxone considered as same category? Many individuals on chronic long-term pain treatment may also be prescribed Narcan to prevent unintentional overdose for individuals prescribed opioids for chronic pain. They may not necessarily be treated for OUD.**
We did not consider naloxone dispensed separately from buprenorphine (e.g. as Narcan), but included only the combination buprenorphine/naloxone product, which is the buprenorphine product indicated for OAT in Ontario. We have clarified this in the methods.

**Revised Text (Methods – Exposure):**
*First, we identified individuals with a dispensed prescription for methadone or the buprenorphine/naloxone combination product overlapping the 14 days prior to or including the enrolment termination date,*… **How did you handle missing data?**
There was no missing data for age or sex. For categories with missing data, the individuals with missing information were categorized separately and included in models and presented in tables.  **6. Results: More information is needed on the healthcare system. What is the difference between a fee for service physician and a PEM?
Discussion: There is now information on the question above about fee-for-service. I think it would be very beneficial to provide an overview of the healthcare system in which the study occurred (Ontario) for those unfamiliar with Ontario's system. This should occur in the introduction.**
We have provided more detail on primary care provision in Ontario in the Introduction of our revised manuscript. As the reviewer mentions here and in comment #2 above, we agree that it was beneficial for the reader to have this detailed information early in the manuscript to help contextualize the findings.

**Inserted Text (Introduction):** *See response to #2 above.*

**References:**

1.	Tao W, Agerholm J, Burstrom B. The impact of reimbursement systems on equity in access and quality of primary care: A systematic literature review. BMC Health Serv Res. 2016;16(1):542. doi: 10.1186/s12913-016-1805-8. PubMed PMID: 27716250.

2.	Hutchison B, Glazier R. Ontario's primary care reforms have transformed the local care landscape, but a plan is needed for ongoing improvement. Health Aff (Millwood). 2013;32(4):695-703. Epub 2013/04/10. doi: 10.1377/hlthaff.2012.1087. PubMed PMID: 23569049.
