# Supplementary material for: Inequities in access to primary care among opioid recipients in Ontario, Canada: A population-based cohort study
Source: PLoS Med. 2021 Jun 1;18(6):e1003631. doi: 10.1371/journal.pmed.1003631 (PMC8168863; doi:10.1371/journal.pmed.1003631)
Supplement: Attachment — Submitted filename: Response to Reviewer Comments - March 25 2021.docx [file pmed.1003631.s009.docx]

**COMMENTS FROM ACADEMIC EDITOR:**

**Note on the first point of Reviewer 4: The editors do not require changes to be made due to space limitations.**
Thank you for noting this. In line with this recommendation, we have not made the suggested changes by Reviewer 4.

**1.Data availability statement: Please update the link to:** [**https://www.ices.on.ca/DAS**](https://www.ices.on.ca/DAS)
This change has been made.

**2. Analysis plan: Thank you for including the prospective analysis plan as a supporting information file. In the document, it seems that changes made to the plan (such as those added in response to peer review) are indicated in bold type, with rationale. Please note this explicitly at the beginning of the document, and please also note changes made to the prospective analysis plan in the Methods section, with rationale.**
This change has been made.

Analysis Plan: added row to table:
*Note: Any changes to the original protocol are underlined and prefaced using the heading “Post-hoc after reviewer comments:”*

Methods:
*In two post-hoc sensitivity analyses, we refit models for the primary outcome among individuals whose physician ended the patient enrolment (to investigate whether findings were consistent when provider loss was not the patient’s decision), and stratified by OAT type (because of different patient and clinical characteristics between methadone and buprenorphine/naloxone).*

**3. Abstract: Methods and Findings: Please mention the setting (Ontario, Canada) more explicitly, and please provide relevant demographic/ enrollment model characteristic information for the study population.**
These changes have been made:

Abstract Methods:
*We conducted a retrospective cohort study using linked administrative data in Ontario, Canada whose enrolment with a physician practicing in a primary care enrolment model was terminated between January 2016 and December 2017.*

Abstract Results:
*In general, OAT recipients were younger (median age 36) than those receiving long-term OPT (59 years) and those with no recent prescription opioid exposure (44 years). In all exposure groups, the majority of individuals had their physician terminate their enrolment (range 78.1% to 88.1%).*

**4. Abstract: Methods and Findings: Please provide p values in addition to the 95% CIs given for the comparison between OAT recipients and non-recipients with likelihood of finding a provider, as well as the comparison for OPT recipients. For the secondary analyses comparing the PCP-loss period with the prior year, please also include the p values for these results.**

These changes have been made:

Abstract Methods and Findings:

*In the primary analysis, as compared to those not receiving opioids, OAT recipients were significantly less likely to find a primary care provider within 1 year (adjusted hazard ratio [aHR] 0.55, 95% confidence interval [CI] 0.50-0.61, p<0.0001). We observed no significant difference between long-term OPT and opioid unexposed individuals (aHR 0.96; 95% CI 0.92-1.01, p=0.12). In our secondary analysis comparing the period of PCP loss to the year prior, we found that rates of ED visits were elevated among people not receiving opioids (adjusted rate ratio (aRR) 1.20, 95% CI 1.18-1.22, p<0.0001) and people receiving long-term OPT (aRR 1.37, 95% CI 1.28-1.48, p<0.0001).*

**5. Abstract: Conclusions: For the first sentence, we would suggest a summary of the main findings of the study; beginning with the phrase "In this study, we observed ..." may be useful.**
This change has been made.

Abstract Conclusion:
*In this study, we observed gaps in access to primary care among people who receive prescription opioids, particularly among OAT recipients.*

**6. Author summary: What do these findings mean? In the first bullet point, we suggest changing “...which is likely influenced by...” to “...which may be influenced by...”**
This change has been made.

Author Summary:
*Gaps in access to primary care exist for people with opioid use disorder and chronic pain, which may be influenced by stigma, discrimination, and financial disincentives within the healthcare system.*

**7. Throughout the text: Please place the in-text reference brackets before, rather than after, the sentence punctuation, like this [1]. When multiple references are listed, please remove spaces within the brackets.**
This change has been made.

**8. Methods: Please add the following statement to the Methods: "This study is reported as per the REporting of studies Conducted using Observational Routinely-collected Data (RECORD) extension of the Strengthening the Reporting of Observational Studies in Epidemiology (STROBE) guideline (S1 Checklist)."**
This statement has been added to the *Study Design and Setting* section of the methods.

**9. Results: Please include p values in addition to the CIs associated with the following results: “After adjusting for potential confounders, OAT recipients were significantly less likely to secure a primary care provider within one year compared to opioid unexposed individuals (adjusted hazard ratio [aHR] 0.55, 95% confidence interval [CI] 0.50 to 0.61).”**

These changes have been made:

Results:

*After adjusting for potential confounders, OAT recipients were significantly less likely to secure a primary care provider within one year compared to opioid unexposed individuals (adjusted hazard ratio [aHR] 0.55, 95% confidence interval [CI] 0.50 to 0.61, p<0.0001).*

**10. Results: Please provide both p values and 95% CIs for all results presented in the text.**

These changes have been made:

Results:

*After adjusting for potential confounders, OAT recipients were significantly less likely to secure a primary care provider within one year compared to opioid unexposed individuals (adjusted hazard ratio [aHR] 0.55, 95% confidence interval [CI] 0.50 to 0.61, p<0.0001). Results for OAT recipients were consistent across all sensitivity analyses. We observed no significant difference in subsequent primary care attachment between long-term OPT recipients and opioid unexposed individuals (aHR 0.96; 95% CI 0.92 to 1.01, p=0.12). However, when considering attachment to a PEM only (aHR 0.85, 95% CI 0.80 to 0.90, p<0.0001) or attachment to a PEM or CHC (aHR 0.87, 95% CI 0.82 to 0.92, p<0.0001), we observed a significantly lower hazard of primary care attachment among long-term OPT recipients compared to opioid unexposed individuals (Table 2).*

*In our secondary analysis, we found that rates of ED visits increased among opioid unexposed individuals (adjusted rate ratio (aRR) 1.20, 95% CI 1.18 to 1.22, p<0.0001) and long-term OPT recipients (aRR 1.37, 95% CI 1.28 to 1.48, p<0.0001) during the period of provider loss. Although OAT recipients did not experience an increased rate of ED visits during their period of provider loss (adjusted rate ratio (aRR) 1.09, 95% CI 0.97 to 1.22, p=0.13), they visited the ED at a much higher rate than opioid unexposed individuals both prior to and following their loss of primary care provider (Table 3; Figure S2).*

**11. Funding and acknowledgement section: Please remove the funding information from the main text. Instead, please include all accurate funding information in the Financial Disclosures section of the manuscript submission form.**
This change has been made.

**12. Please remove the “Conflicts of interest” section from the main text and ensure this information is accurately entered in the “Competing Interests” section of the manuscript submission form.**
This change has been made.

**13. Table 2 and Table 3: Please also provide p values for the adjusted and unadjusted analyses.**

These changes have been made.

**COMMENTS FROM THE REVIEWERS**

**Reviewer #1: The authors have addressed all my points.**

We are glad we were able to address all of your points

**Reviewer #3: The authors have been highly responsive to review and have submitted a stronger paper. I have no further comments.**
We thank the reviewer for their suggestions, which we agree have strengthened the paper.

**Reviewer #4: The authors did a very nice job of thoughtfully addressing the feedback from the editors and previous reviews. The overview on healthcare in Ontario in the Introduction was very helpful to orient the reader to the system being examined in the study; thank you for including this. I applaud the addition of briefly mentioning both stigma and financial disincentives in reason why some patients have difficulty securing primary care, as well as the additional references. Thank you for providing more information on the rational for excluding patients enrolled in palliative care, as well as clarification on naltrexone and buprenorphine/naloxone. The author summary is a clear and concise reflection of the study content.**
We thank the reviewer for their positive comments.

**Would defer to editor on this regarding space: On p. 31 - consider whether these paragraphs on exclusion criteria and methods may be better presented in a table? (This may only be necessary if space is an issue).**
Based on advice from the editor, we have not made this change.

**Within the discussion, the authors do a good job of highlighting that there is much work to be done to improve the healthcare system for patients who use opioids and OAT, especially addressing stigma and discrimination. While it is not necessary to provide an in-depth analysis of what can be done about these matters due to space, I wonder if the authors could provide a brief summary of what (from a public policy standpoint as well as potentially continuing education, within training programs, etc.) might be done to mitigate the effects of these disparities that are identified for people with OUD.**
We thank the reviewer for this suggestion. We have now included a brief summary of what can be done in this space.

Discussion:
*Future efforts are needed to more fully understand the patient and provider-level factors that contribute to these gaps, including financial disincentives within reimbursement models, addressing stigma and discrimination within the healthcare system, and creating safe environments for marginalized populations. For example, physician remuneration policies should be reviewed to ensure that they do not reinforce structural barriers and discrimination, and physician education should elucidate how stigmatizing language and behaviors can harm marginalized populations when they interact with their healthcare providers.*
